# A Singular Case of Death Subsequent to Delivery

**Published:** 1856-10

**Authors:** O. C. Gibbs

**Affiliations:** Frewsburg, Chautauque Co., N. Y.


*A singular Case of Death subsequent to delivery.* By O. C. Gibbs, M. D., of Frewsburg, Chautauque Co., N. Y.

In the Medical Examiner for August, Dr. Gemmill communicates a "singular case of death, occurring twenty-two days after instrumental delivery," which brings to my mind a case presenting several points of resemblance, which came under my observation nearly two years since. As the case presents some points of interest I forward you a brief report of it.

November 1st, 1854, I was called to see Mrs. A., and, on arriving at her residence, found her in labor at full term. Mrs. A. was about 35 years of age, and had given birth to a living child at full term some twelve years previously, since which time she had had four premature deliveries at seven months. Labor progressed favorably and she gave birth to a healthy female child, about six hours after I was called to the case.

After her first confinement she had suffered very much from *phlegmasia dolens*, in consequence of which she was fearful that some untoward symptoms would interfere with speedy recovery in the present instance. I saw her occasionally for the first ten days, but as no symptoms requiring treatment presented themselves I did not further enquire after the case.

November 20th, about three weeks from the time of confinement, I was again summoned to see her, about ten o'clock in the evening. The husband, who came for me, said that he did not think there was any necessity for alarm, as probably the symptoms were wholly hysterical. On arriving at the bedside of my patient, I found her laboring under the greatest dyspnœa. The number of respirations per minute was treble that of health, and the countenance was expressive of very great distress. Air seemed to enter the lungs freely, but seemed utterly unavailing for all the purposes for which respiration is accomplished. Any attempt to examine the lungs or heart, seemed to aggravate her distress, and, in the tumultuous manner in which these organs were performing their functions, such examination would doubtless have led to no satisfactory result. The pulse was so rapid that it could not be well counted. From the husband, I learned that she had been apparently doing well, until about an hour before I saw her. She had been sitting up every day for a week or more, and had taken the principal charge of her child. She had suffered somewhat from pain in her legs, but as they were not swollen much, if any, he had not sought my advice in regard to that symptom, and, in fact, for the last few days she had even improved in that particular. She had complained that her lochial discharges were more offensive than usual, but, on the whole, considered herself to be making a fortunate recovery. On the evening of the attack, she had been up until about nine o'clock, had prepared her child and herself for bed, laid down and had gotten nearly to sleep, when the paroxysm of dyspnœa came on.

She lingered about thirty hours, without any abatement of the symptoms, and unaffected by treatment, being perfectly concious until within a few minutes of the last respiratory effort. In this case, as in that reported by Dr. Gemmill, no post mortem examination was permitted, much to my regret. The patient had never complained of heart disease, and, though intimately acquainted with her for years, my attention had never been directed to that organ as subject to abnormal action. I diagnosticated fibrinous concretions or coagulative accumulation within the heart. It is quite possible, I think, that a fibrinous deposit, breaking loose from one of the valves of the heart, had caught in the trunk of the pulmonary artery, thus accounting for some of the peculiar respiratory symptoms. If any of the readers of the Examiner, more fortunate than Dr. Gemmill and myself, have seen the autopsy of a case like the above and under similar circumstances, I trust they will report the result of their examination.

